# Half of all hip and knee arthroplasty patients may be potential day-case candidates: a nationwide register study of 166,730 procedures

**DOI:** 10.2340/17453674.2024.40075

**Published:** 2024-02-23

**Authors:** Christian Bredgaard JENSEN, Anders TROELSEN, Nicolai Bang FOSS, Christian Skovgaard NIELSEN, Martin LINDBERG-LARSEN, Kirill GROMOV

**Affiliations:** 1 Department of Orthopaedic Surgery, Clinical Orthopaedic Surgery Hvidovre (CORH), Copenhagen University Hospital Hvidovre, Hvidovre; 2 Department of Anesthesiology, Copenhagen University Hospital Hvidovre, Hvidovre; 3 Department of Orthopaedic Surgery, Orthopaedic Research Unit (ORU), Odense University Hospital, Odense, Denmark

## Abstract

**Background and purpose:**

The overall potential pool of day-case candidates on a national level in hip and knee arthroplasty is unknown. We aimed to estimate the proportion of hip and knee arthroplasty patients eligible for day-case surgery based on contemporary widely used criteria and determine whether there has been a change in the proportion of eligible patients over time and, secondarily, to investigate the proportion of eligible patients discharged on the day of surgery.

**Methods:**

Based on data from the Danish National Patient Register, we identified all patients undergoing primary unilateral hip or knee arthroplasty from January 2010 to March 2020. Using a modification of day-case eligibility criteria proposed by a national multicenter collaboration, we sorted patients into either day-case eligible or ineligible. A day-case procedure was defined as discharge on the day of surgery.

**Results:**

We included patients comprising a total of 166,730 primary total hip (THA), total knee (TKA), and unicompartmental knee arthroplasty (UKA). 48% (95% confidence interval [CI] 48–49) were eligible for day-case surgery, with a decline from 50% (CI 49–51) in 2010 to 46% (CI 46–47) eligible in 2019. More UKA patients were day-case eligible (55%, CI 54–56) than THA (47%, CI 47–48) and TKA patients (49%, CI 48–49). A maximum of 8.0% (CI 7.4–8.5) of eligible patients were discharged on the day of surgery in 2019.

**Conclusion:**

48% of the Danish hip and knee arthroplasty patients were potential day-case candidates, with a small decline in eligibility from 50% in 2010 to 46% in 2019. Day of surgery discharge among day-case eligible patients peaked at 8% in 2019. Thus, the potential for more day-case surgery seems large.

Day-case procedures, where patients are discharged on the day of surgery, are used increasingly in hip and knee arthroplasty [1,2]. Preceded by years of continuous safe reductions in length of hospital stay, day-case surgery is a next step in potentially optimizing bed occupancy and offering cost reductions [3]. Studies have found that day-case hip and knee arthroplasty is safe. It is widely agreed that patient selection is paramount in securing the safety of day-case procedures [4].

Eligibility criteria for day-case hip and knee arthroplasty vary from center to center, as do the day-case setups [5-7]. A recently published study protocol reports a consensus on day-case eligibility criteria as followed by 8 high-volume arthroplasty centers covering 50% of all hip and knee arthroplasty procedures in Denmark [8]. However, the nationwide potential for day-case hip and knee arthroplasty is unknown. Smaller cohort studies report varying proportions of eligibility ranging from 31–75% of patients [7,9,10]. It has also been suggested that utilization of day-case surgery is low, compared with the total number of eligible patients [9]. Knowing the potential for day-case arthroplasty and the utilization rate in eligible patients is important to provide targets for day-case use, as well as showcasing the magnitude of the impact that optimal day-case use may have.

We aimed to estimate the proportion of patients eligible for day-case hip and knee arthroplasty on a nationwide level and determine whether there has been a change in the proportion of eligible patients over time. Second, we aimed to investigate the proportion of day-case eligible arthroplasty patients discharged on the day of surgery.

## Methods

### Design and setup

Data for this study originated from the Danish National Patient Register (DNPR) and the Danish Civil Registration System (CPR). Our report complies with the REporting of studies Conducted using Observational Routinely-collected Data (RECORD) statement. Previous studies on the use of day-case surgery and the risk of readmission following day-case surgery have been conducted on this cohort [11,12].

In the Danish healthcare system, most hip and knee arthroplasty procedures are performed in publicly funded hospitals. Arthroplasty procedures performed in private hospitals are either publicly funded, insurance funded, or out-of-pocket funded. Public funding for private hospital treatments is available when public hospitals are not able to meet treatment guarantees due to long waiting lists. The primary discharge destination for Danish arthroplasty patients is the patient’s own home, as rehabilitation hospitals or skilled nursing facilities are not used.

### Data sources

We acquired data on all hip and knee arthroplasties performed between January 2010 and March 2020 from the DNPR and CPR. The DNPR is a national administrative database containing data on all hospital visits and admissions of patients to Danish hospitals [13]. Surgical procedure codes in the DNPR are based on the Nordic Medico-Statistical Committee (NOMESCO) classification of surgical procedures (NCSP) [14]. Diagnosis codes are based on the International Classification of Diseases and Related Health Problems 10th Revision (ICD-10).

From the CPR register, we acquired data on the patients’ marital status. We linked data from the DNPR and the CPR register using the civil registration number (in an encrypted format), which is unique and required for each Danish inhabitant.

### Study population

Using surgical codes, we identified total hip arthroplasties (THA: NFB20, NFB30, NFB40), total knee arthroplasties (TKA: NGB20, NGB30, NGB40), and unicompartmental knee arthroplasties (UKA: NGB01, NGB02, NGB11, NGB12) in the DNPR treated in both private and public hospitals. We included only primary unilateral procedures with a diagnosis code of hip or knee osteoarthritis (OA: M16n or M17n) and a registered admission and discharge date. We defined simultaneous bilateral procedures in the following 2 ways: (1) a left and a right procedure on the same patient on the same day or (2) a single procedure coded with either bilateral or left and right. Staged bilateral procedures were included as 2 separate cases.

### Outcome measures

We used the updated criteria for day-case eligibility published by an ongoing Danish multicenter collaboration as the basis for our eligibility criteria [8]. Some criteria are modified to give a similar, but conservative, estimate, as the original criteria cannot be evaluated with the data available through the DNPR and CPR. In the current study we considered patients ineligible for day-case surgery if any of the following criteria were met: (1) age above 80 years, (2) no registered partnership based on data from the CPR, (3) any prior hospital visits with diagnosis codes for any of the following: very severe obesity (BMI > 40), congestive heart failure, diabetes mellitus type 1, hypoglycemic episodes, chronic obstructive pulmonary disease, ischemic heart disease, chronic renal failure, sleep apnea, and (4) any hospital visits within 90 days prior to surgery with diagnosis codes for any of the following: myocardial infarction, transient ischemic attack or stroke, repeated falls. As a surrogate measure of falls we also considered patients receiving computed tomography of the brain within 90 days of surgery ineligible for day-case surgery. All diagnosis or procedure codes used to define the above criteria are listed in Table 1 (see Supplementary data).

A day-case surgery was defined as the patient being operated on and discharged on the same date. Readmissions occurring less than 4 hours after discharge from the primary surgical admission were coupled with the primary admission [11,15]. Length of stay (LOS) then accounts for the total number of nights admitted to hospital during the coupled admissions.

### Statistics

Continuous variables were evaluated for normality using histograms and Q–Q plots. Normally distributed variables were presented as mean and standard deviation (SD), and non-normally distributed variables were presented as median and interquartile range (IQR). For categorical outcome variables we presented proportions as percentages with 95% confidence intervals (CI) calculated using the binomial exact calculation (Clopper-Pearson method [16]).

The statistical analyses were performed using R version 4.1.3 (R Core Team, 2022; R Foundation for Statistical Computing, Vienna, Austria) and R Studio version 2022.07.2 (R Studio Team, 2022).

### Ethics, funding, data sharing, and disclosures

This study was approved by the Knowledge Centre on Data Protection Compliance in the Capital Region of Denmark (approval nr. P-2021-132). According to Danish law, register-based studies do not require approval from national research ethical committees and informed consent was waived. No funding was received specifically for this study. Data sharing is not allowed due to Danish law. CBJ has received PhD funding from a grant from the Novo Nordisk Foundation unrelated to this study. KG and AT have received research support and speaker fees from Zimmer Biomet, and AT has received research support from Pfizer. NBF has received speaker fees from Masimo Corporation and Edwards Lifesciences. All the above conflicts are unrelated to this study. Complete disclosure of interest forms according to ICMJE are available on the article page, doi: 10.2340/17453674.2024.40075

## Results

**Figure 1:**
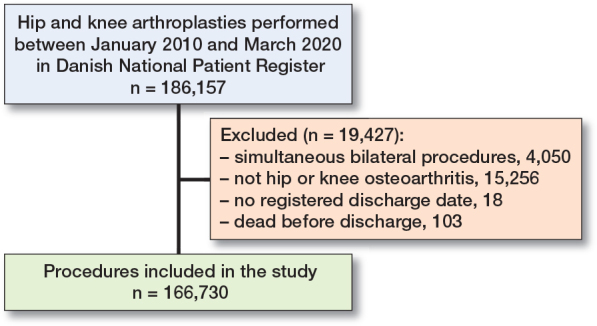
Patient flowchart with exclusions.

186,157 procedures were identified: 4,050 were bilateral procedures, 15,256 had surgical indications other than hip or knee OA, 18 had no registered discharge date, and 103 died prior to discharge. The final cohort consisted of 166,730 primary hip and knee arthroplasty patients (86,011 THAs, 70,281 TKAs, and 10,438 UKAs) (Figure 1). Based on the day-case eligibility criteria, 80,622 (48%, CI 48–49) patients were eligible for day-case surgery. For THA, TKA, and UKA patients, respectively, 40,638 (47%, CI 47–48), 34,207 (49%, CI 48–49), and 5,777 (55%, CI 54– 56) patients were eligible for day-case surgery. Day-case eligible patients were younger and more often male compared with ineligible patients (Table 2). The 3 most frequent reasons for being ineligible for day-case surgery were having no registered partnership (75%), being older than 80 years of age (23%), and history of a hospital visit with a diagnosis code for ischemic heart disease prior to surgery (16%) (Table 3). Of the day-case ineligible patients, 44,671 patients (52%) were only ineligible due to having no registered partnership. Acute onset of severe illness, such as stroke, within 90 days of surgery rarely made patients ineligible.

**Table 2:** Patient characteristics for eligible and ineligible patients. Values are n (%) unless otherwise specified

Factor	Eligible patients n = 80,622	Ineligible patients n = 86,108
Male sex	38,088 (47)	33,114 (39)
Age, mean (SD)	65.8 (8.6)	70.4 (11.1)
Public hospital	74,394 (92)	81,808 (95)
Year of surgery		
2010–2013	30,417 (38)	31,380 (36)
2014–2017	31,294 (39)	33,304 (39)
2018–2020	18,911 (24)	21,424 (25)

SD = standard deviation. Year of surgery was stratified in accordance with previously conducted studies on the study cohort.

**Table 3:** Distribution of criteria making hip and knee arthroplasty patients ineligible for day-case surgery. Values are n (%)

Factor	Ineligible patients n = 86,108
Age < 18 or > 80	20,155 (23)
No registered partnership	64,665 (75)
Body mass index > 40	372 (0.4)
Within 90 days preoperatively	
Myocardial infarction	34 (0.0)
Transient ischemic attack	88 (0.1)
Stroke	84 (0.1)
Repeated falls	67 (0.1)
Computed tomography of the brain	745 (0.9)
Congestive heart failure	3,011 (3.5)
Diabetes mellitus type 1	1,004 (1.2)
Hypoglycemic episodes	282 (0.3)
Chronic obstructive pulmonary disease	3,967 (4.6)
Chronic renal failure	1,568 (1.8)
Ischemic heart disease	13,390 (16)
Sleep apnea	4,800 (5.6)

Ineligible patients can have multiple reasons for ineligibility.

Percentages display the proportion of ineligible patients fulfilling each specific ineligibility criterion. Definition of each criterion can be seen in Table 1 (see Supplementary data).

**Figure 2:**
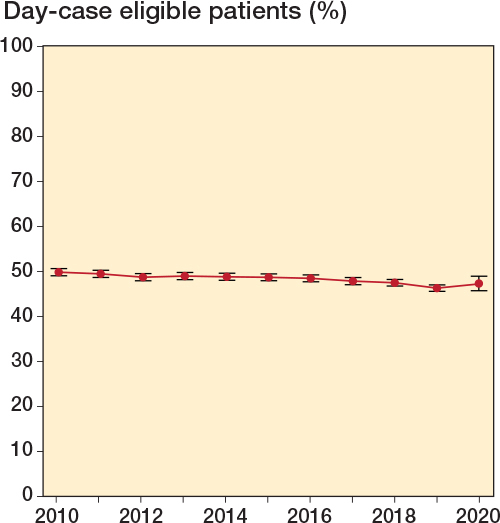
Proportion of day-case eligible arthroplasty patients over time from 2010–2020. Error bars indicate 95% confidence intervals. The year 2020 has data only from January until end of March.

**Figure 3:**
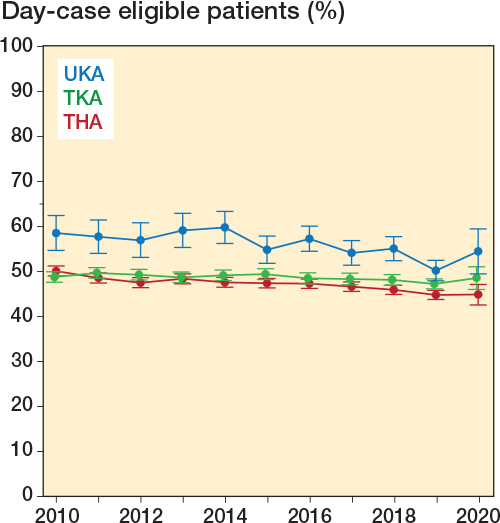
Proportion of total hip arthroplasty (THA), total knee arthroplasty (TKA), and unicompartmental knee arthroplasty (UKA) patients considered eligible for day-case surgery over time, see Figure 2.

The proportion of eligible patients declined from 50% (CI 49–51) in 2010 to 46% (CI 46–47) in 2019 (Figure 2). This trend was observed across all arthroplasty types (Figure 3). The proportion of patients between 18 and 80 years of age declined from 89% (CI 88–89) in 2010 to 87% (CI 87–87) in 2019. Similarly, the proportion of patients without any medical issues making them ineligible for day-case surgery declined from 86% (CI 86–87) in 2010 to 85% (CI 85–86) in 2019 .

**Figure 4:**
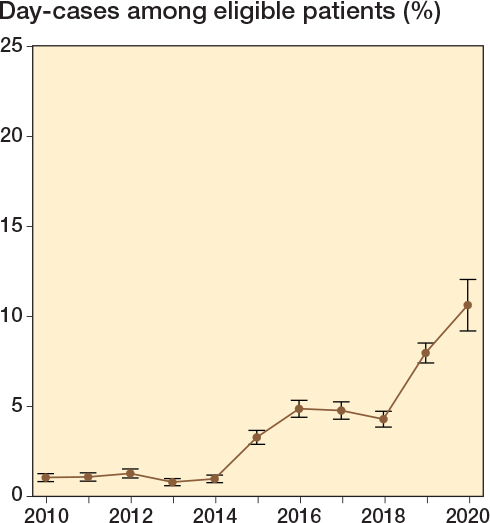
Proportion of discharge on the day of surgery for day-case eligible arthroplasty patients over time, see Figure 2.

**Figure 5:**
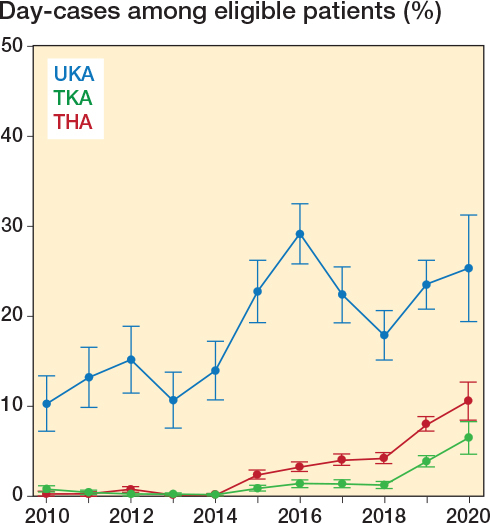
Proportion of discharge on the day of surgery for day-case eligible THA, TKA and UKA patients, see Figure 2 and 3 for abbreviations.

Day-case eligible patients were increasingly discharged on the day of surgery from 2015 at 3.3% (CI 2.9–3.7), reaching 8.0% (CI 7.4–8.5) in 2019 (Figure 4). The proportion of eligible patients discharged on the day of surgery was highest for UKA (Figure 5).

## Discussion

We aimed to estimate the proportion of patients eligible for day-case hip and knee arthroplasty and to investigate the proportion of day-case eligible arthroplasty patients discharged on the day of surgery. We found that from 2010–2020 hip and knee arthroplasty patients were eligible for day-case surgery in 48% of cases. More UKA patients were eligible for day-case surgery (55% vs. 47% of THAs and 49% of TKAs). We found a slight decrease in the proportion of patients aged < 80 years (from 89% to 87%) and patients with no medical issues (from 86% to 85%). While these declines are likely not of major clinical relevance, they may partly explain the decrease in day-case eligibility from 50% in 2010 to 46% in 2019. The utilization of day-case surgery increased over time from 3.3% in 2015 to 8.0% of day-case eligible patients in 2019.

A previous investigation from 2 centers in Denmark found that 54% of THA and TKA patients (n = 557) were eligible for day-case surgery using comparable criteria [9]. A study based on Canadian TKA patients (n = 400) found that only 31% were day-case candidates; however, 55% were potential candidates if modifiable reasons for ineligibility were addressed [7]. Conversely, a French study found that 75% of patients were eligible for day-case surgery [10] when eligibility was evaluated on a patient-by-patient basis with no predefined eligibility criteria. While differences in demographics between national arthroplasty populations have been reported [17,18], the applied criteria of day-case eligibility likely accounted for the reported differences in potential candidates for day-case surgery. In previous studies, day-case eligibility criteria were commonly based on cardiovascular disease (beyond hypertension), pulmonary disease, insulin-dependent diabetes, sleep apnea, or anemia [5,19,20]. However, regardless of the criteria used, the number of potential day-case candidates exceeded the reported national utilization of day-case surgery by a large margin [1,11,21].

When applying our eligibility criteria, we found that the pool of day-case candidates decreased slightly over time, primarily due to increasing age and comorbidity in the arthroplasty population, as previously evaluated in this nationwide cohort [11]. The growing elderly population has increased the occurrence of multimorbidity [22], but improved living conditions and medical advances might also create a population of healthy elderly patients. While comorbidity, age, and risk of complications are complexly associated, appropriate selection of day-case candidates based on comorbidity alone might be sufficient to exclude at-risk patients and not exclude otherwise healthy elderly patients [23]. Other clinical evaluations, such as the Clinical Frailty Scale, may be used to summarize medical and age-related issues in regard to day-case eligibility [8], instead of strict age criteria.

A major reason for ineligibility was having no registered partnership. However, patients could still have had an adult at home for support, despite having no registered partnership. Other studies have also reported that non-medical issues, such as insufficient support at home or large hospital-to-home distance, accounted for a notable portion of day-case ineligible patients [7]. One study reported that no help at home and large distance to the hospital resulted in 5% and 12% of patients being ineligible for day-case surgery, respectively. Another study reported that safe discharge on the day of surgery was still a possibility for such patients, when discharged to a rehabilitation center [10]. Whether this is a potential solution still offering significant cost reductions requires further research.

### Strengths

First, this study comprises a large, nationwide patient population, as this eliminates potential patient selection at a specific surgical center or region. Second, the day-case eligibility criteria we have applied are already used in a clinical setting at most major arthroplasty centers in Denmark. And while we had to modify the day-case criteria, we chose the most conservative alternative criteria to not overestimate the potential pool of day-case candidates. For example, regarding the criterion on sleep apnea requiring mechanical treatment, we excluded any patients seen with a diagnosis of sleep apnea, as data on the use of mechanical treatment was not available. The most prevalent reason for ineligibility was having no registered partnership. This criterion was used as a conservative surrogate measure of having insufficient support at home, utilizing the data available. For the criterion on chronic obstructive pulmonary disease (COPD) with home oxygen, we excluded any patients seen with a diagnosis of COPD within the last 10 years.

### Limitations

The reliance on database codes for identifications of day-case eligibility criteria are a limitation. A direct transfer of the criteria was not possible, because the data needed to evaluate certain criteria is not available in the DNPR or CPR registers. Also, any known major differences in demographics between various arthroplasty populations must be considered when interpreting our results. However, using a complete national cohort improves generalizability in comparable populations.

### Conclusion

48% of the Danish hip and knee arthro-plasty patients were potential day-case candidates, with a small decline in eligibility from 50% in 2010 to 46% in 2019. Day of surgery discharge among day-case eligible patients peaked at 8% in 2019.

In perspective, our study provides a conservative benchmark for day-case use based on complete nationwide data. The potential for more day-case surgery seems large.

### Supplementary data

Table 1 is available as supplementary data on the article page, doi: 10.2340/17453674.2024.40075

## Supplementary Material



## Notes

CBJ and KG had full access to all data in the study and take responsibility for the integrity of the data and the accuracy of the data analysis. Study concept and design: CBJ, AT, MLL, and KG. Acquisition, analysis, and interpretation of data: all authors. Drafting of the manuscript: CBJ and KG. Critical revision of the manuscript for important intellectual content: all authors.

Handling co-editor: Bart Swierstra

*Acta* thanks Martijn Schotanus and Annette W-Dahl for help with peer review of this manuscript.
