# Supplementary material for: Half of all hip and knee arthroplasty patients may be potential day-case candidates: a nationwide register study of 166,730 procedures
Source: Acta Orthop. 2024 Feb 23;95:147–51. doi: 10.2340/17453674.2024.40075 (PMC10886217; doi:10.2340/17453674.2024.40075)
Supplement: Supplementary file 1 [file ActaO-95-40075-s1.pdf]

Supplementary data

Table 1. Day-case eligibility criteria used by the Centre for Fast-track Hip and Knee Replacement collaboration and the corresponding register-based adaptation used in the current study

Center for Fast-track Hip and Knee Replacement day-case criteria	Corresponding criteria used in the current study
Unilateral elective primary THA, TKA or UKA	Surgical procedures codes:<br>THA (NFB20, NFB30, NFB40)<br>TKA (NGB20, NGB30, NGB40)<br>UKA (NGB01, NGB02, NGB11, NGB12)<br>Only procedures performed with a diagnosis code for hip or knee osteoarthritis (M16n or M17n) were included<br>All simultaneous bilateral procedures are excluded<br>No revision procedures codes are included
Age 18–80	Age 18–80
Acute myocardial infarction, cerebrovascular accident, transient ischemic attack, or coronary atherosclerotic disease within last 3 months	Any hospital visits with the following diagnosis codes within the 90 days prior to surgery:<br>Myocardial infarction: I21n, I22n, I23n, I24n<br>Cerebrovascular accident: I60n, I61n, I62n, I63n, I64n<br>Transient ischemic attack: G45n
Unstable ischemic heart disease	Any hospital visits with the following diagnosis codes within the 10 years prior to surgery:<br>Ischemic heart disease: I20n, I25n
Ejection fraction < 40%	Any hospital visits with the following diagnosis codes within the 10 years prior to surgery:<br>Heart failure: I50n, I11.0, I13.0, I13.2
Glomerular filtration rate < 60 mL/min/1.73 m^2	Any hospital visits with the following diagnosis codes within the 10 years prior to surgery:<br>Chronic kidney disease (CKD): N18n
Chronic obstructive pulmonary disease with home oxygen	Any hospital visits with the following diagnosis codes within the 10 years prior to surgery:<br>Chronic obstructive pulmonary disease: J44n
Insulin-dependent diabetes mellitus	Any hospital visits with the following diagnosis codes within the 10 years prior to surgery:<br>Diabetes type I: E10n<br>Hypoglycemic episodes: E16.0, E16.2
Sleep apnea requiring mechanical treatment	Any hospital visits with the following diagnosis codes within the 10 years prior to surgery:<br>Sleep apnea: G17.3
2 or more falls within last 3 months	Any hospital visits with the following diagnosis codes within the 90 days prior to surgery:<br>Repeated falls: R29.6<br>Computed tomography scan of the brain: UXCA00 (Danish National Patient Register examination code for radiologic examinations).
Body mass index (BMI) < 18.5 or > 40	Any hospital visits with the following diagnosis codes within the 10 years prior to surgery:<br>Obesity due to excess calories: E66.0E, E66.0F, E66.0G, E66.0H<br>(Danish National Patient Register adaptation of ICD-10, with added letters E, F, G, H indicating: BMI 40–44.9, BMI 45–49.9, BMI 50–54.9, and BMI 55+)
Not interested in discharge on day of surgery and Clinical Frailty Scale ≥ 4	No appropriate diagnosis code or procedure code adaptation of these criteria were available

Diagnosis codes are from the Danish National Patient Register based on the International Classification of Diseases and Related Health Problems—10th revision (ICD-10). Surgical procedure codes are based on the Nordic Medico-Statistical Committee (NOMESCO) classification of surgical procedures (NCSP). Radiological examinations were identified using the Danish National Patient Register examination codes for radiologic examinations. An "n" indicates any following subclassification number related to the ICD-10 codes.
